# Adult-onset and late-onset multiple sclerosis in older adults in a Finnish university hospital

**DOI:** 10.1007/s10072-025-08756-w

**Published:** 2026-01-08

**Authors:** Kimi Ahtinen, Iiro Korhonen, Hanna Kuusisto, Ilkka Rauma

**Affiliations:** 1 Faculty of Medicine and Health Technology, Tampere University, Tampere, Finland; 2 Neural Medicine Responsibility Sector, Department of Sensory, Neural and Musculoskeletal Medicine, Neurocenter Finland, Tampere Brain and Mind, Wellbeing Services County of Pirkanmaa, Tampere University Hospital, Tampere, Finland; 3 Department of Health and Social Management, Faculty of Social Sciences and Business Studies, University of Eastern Finland, Kuopio, Finland; 4 Department of Neurology, Tampere University Hospital, PO BOX 2000, Tampere, FI-33521 Finland

**Keywords:** Aged, Clinical course, Multiple sclerosis, Onset age, Retrospective studies

## Abstract

**Background:**

The clinical characteristics of multiple sclerosis (MS) in individuals aged 65 years and older remain scarcely studied. This retrospective study compared late-onset MS (LOMS, onset ≥ 50 years) and adult-onset MS (AOMS, onset 18–49 years) in older adults with MS (OAwMS) aged ≥ 65 years.

**Methods:**

Data of subjects aged ≥ 65 years with a confirmed MS diagnosis and a recorded age at onset were collected from the Finnish MS registry at Tampere University Hospital, excluding individuals with paediatric-onset MS. Demographics, disease-modifying therapy (DMT) use, relapse history and Expanded Disability Status Scale (EDSS) scores were compared between LOMS and AOMS groups.

**Results:**

The cohort included 248 subjects (41 LOMS, 207 AOMS). Primary progressive MS (PPMS) was more common in LOMS when compared to AOMS (41.5% vs. 12.6% at data acquisition, *p* < 0.001). Among subjects with a history of relapsing-remitting MS (RRMS) (32 LOMS, 178 AOMS), DMT had been used by 25.0% and 27.5% of subjects with LOMS and AOMS, and relapses after age 65 occurred in 18.8% and 7.3% of subjects with LOMS and AOMS, respectively. Subjects with LOMS discontinued DMT at an older age when compared to those with AOMS (mean 64.0 vs. 58.8 years, *p* = 0.04). No age-related differences in EDSS were observed after age 65.

**Conclusions:**

PPMS was more common among subjects with LOMS. Among subjects with a current or previous diagnosis of RRMS, occasional relapses were observed after age 65 in both groups, though multiple relapses were rare. These findings support the need for individualised care of OAwMS.

## Introduction

Multiple sclerosis (MS) is an inflammatory and neurodegenerative disease affecting the central nervous system (CNS) [1, 2]. Most people with MS (pwMS) experience their initial symptoms between the ages of 18 and 49, and are categorised as having adult-onset MS (AOMS) [3]. In approximately 5% of all pwMS, the disease manifests after the age of 50, which is the most commonly used cut-off age for late-onset MS (LOMS) [3]. Some studies have also used a cut-off age of 60 years to define very late-onset MS (VLOMS) [4, 5].

The clinical course of LOMS may differ from that of AOMS. LOMS more often presents initially with motor deficits and is associated with an increased risk of progressive MS [3, 6]. The cognitive outcomes in people with LOMS have also been demonstrated to be worse than in AOMS [7]. Although relapses are typically rare in older adults with MS (OAwMS, aged ≥ 65 years), evidence regarding relapse patterns in LOMS remains limited [5, 8, 9].

As the life expectancy of pwMS is now approaching that of the general population, MS is becoming increasingly prevalent among older adults [10]. Aging is associated with a decline in both innate and adaptive immune function, which may increase susceptibility to infections and influence drug safety [11]. This process, often referred to as immunosenescence, reduces the body’s capacity to mount immune responses, but also leads to increased neurodegeneration and reduced remyelination capacity, potentially affecting the clinical course of MS in older individuals [12, 13].

Disease-modifying therapies (DMTs) have brought stability to the lives of many pwMS. However, clinical studies have almost exclusively excluded patients over 55 years, resulting in a lack of research regarding the treatment of OAwMS. A meta-analysis found that none of the currently available DMTs demonstrated efficacy beyond a mean age of 53 years [14]. However, emerging real-world evidence suggests that age-related exclusion from treatment may be unwarranted in patients presenting with inflammatory activity [15]. The optimal treatment approach for individuals with LOMS remains undetermined. Experts have suggested broadening the age range of clinical trials and gathering real-world data to support evidence-based care for the aging MS population [16].

In the era of modern DMTs, where both MS prognosis and life expectancy have improved significantly [17], new research questions have emerged. Since the clinical presentation of MS may vary with age, it is essential to understand the course of LOMS in the long term when compared to that of AOMS. This study aimed at investigating the clinical course of MS among older adults aged 65 years or older with either AOMS or LOMS.

## Methods

This retrospective study (R19613S) was conducted at Tampere University Hospital (TUH) in the Wellbeing Services County of Pirkanmaa, Finland. The clinical characteristics of the overall cohort on which this secondary analysis is based has been published previously [18]. The catchment area of TUH includes approximately 500,000 inhabitants, representing approximately 10% of the Finnish population. The Finnish MS registry was used to identify all adults aged 65 years or older living with either AOMS (defined as disease onset at age 18–49) or LOMS (defined as disease onset at age ≥ 50) treated at TUH.

Demographic and clinical data were extracted with a cutoff date of 5 December 2023. Prior to data extraction, the researchers reviewed all registry entries and patient records to identify and correct missing or potentially inaccurate data. The variables used in our analyses included: dates of birth, disease onset, MS diagnosis and data acquisition; sex (as reported in the Population Information System); smoking status; cerebrospinal fluid (CSF)-specific oligoclonal bands; MS type at diagnosis and at data acquisition; DMT history; relapses; and Expanded Disability Status Scale (EDSS) scores after age 65. MS type was based on registry coding done by treating neurologists, who diagnose relapsing-remitting MS (RRMS) or primary progressive MS (PPMS) based on established diagnostic criteria [19–22]. Although the diagnosis of secondary progressive MS (SPMS) is typically made retrospectively when disability progression occurs in the absence of disease activity, the registry includes an algorithm that facilitates early recognition of SPMS. Clinical reassessment of disease type was not performed in this study.

Statistical analyses were performed using SPSS for Windows (version 29) and MedCalc. Continuous variables were summarised using means and standard deviations (SDs) or medians and interquartile ranges depending on data normality, which was assessed using the Shapiro-Wilk test. Categorical variables were presented frequencies and proportions. Comparisons between AOMS and LOMS were conducted using independent samples t-tests or Mann-Whitney U tests for continuous variables, and χ^2^ tests or Fisher’s exact tests for categorical variables. DMT use and relapse data were analysed and reported for subjects with a current or previous diagnosis of RRMS.

Relapses were defined as episodes of new or increasing neurologic dysfunction occurring in the absence of fever or infection and based on clinical documentation by neurologists. Annualised relapse rates (ARRs) were calculated per patient excluding the onset symptom and compared using generalised linear models with age as covariate. Time to first relapse was analysed using Cox regression and reported as hazard ratios (HRs) with 95% confidence intervals (CIs). Both ARR and time to first relapse were calculated from each subject’s 65th birthday or MS diagnosis whichever occurred last. Given the limited sample size, propensity score methods were not used.

The study was conducted in accordance with the Finnish Act on Medical Research [23] and guidelines in non-medical research [24]. As this was a retrospective study with no direct patient contact, informed consent was not required. According to the Act on Medical Research, the guidelines in non-medical research as well as the guidelines of the Research Ethics Committee of the Tampere University, ethical approval is not required for studies involving adults when there is no physical intervention or exposure to exceptionally strong stimuli or sensitive issues. TUH has a Research Ethics Committee which has a mandate to give opinion only on medical studies, as defined in the Act on Medical Research [23].

## Results

**Fig. 1:**
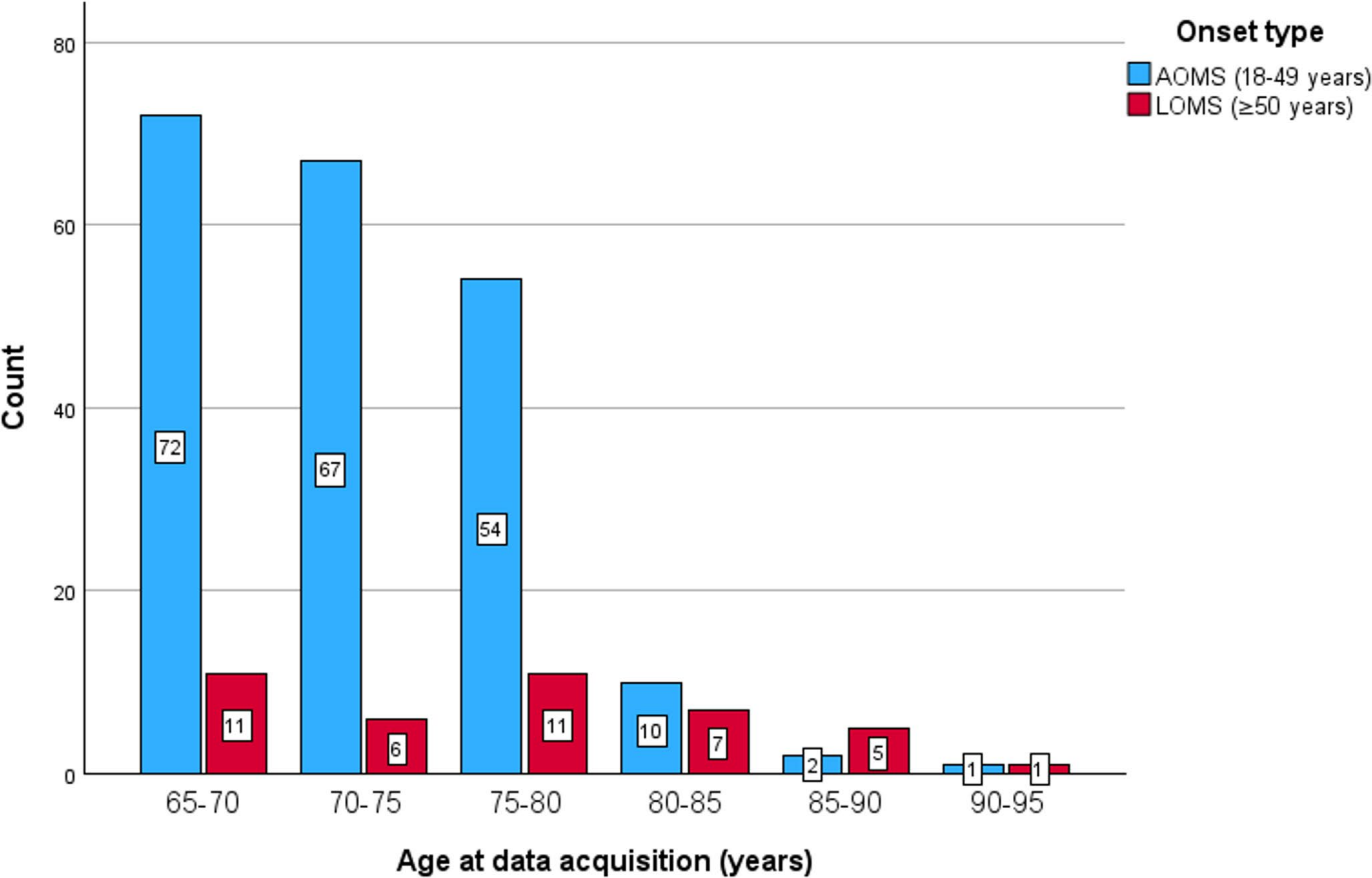
Age distribution at data acquisition. AOMS, adult-onset multiple sclerosis; LOMS, late-onset multiple sclerosis

The study cohort included 248 OAwMS, of whom 207 (83.5%) had AOMS and 41 (16.5%) had LOMS. Demographic details are presented in Table 1, and age distributions at data acquisition in Fig. 1. Eight subjects (3.2%) had symptom onset after the age of 60.

Subjects with LOMS had a higher mean age at disease onset, diagnosis and data acquisition (Fig. 1) compared to those with AOMS (Table 1). Consequently, the mean follow-up duration after age of 65 was longer among subjects with LOMS. No significant differences were observed between the groups in terms of sex, smoking status or the presence of CSF-specific oligoclonal bands in the initial CSF sample (Table 1).

RRMS was the most common initial disease type in both groups, but it was more prevalent among subjects with AOMS (Table 1). At the time of data acquisition, progressive MS (either SPMS or PPMS) was common in both groups (131/187 subjects with AOMS [70.1%] and 29/37 subjects with LOMS [78.4%], *p* = 0.31). At data acquisition, SPMS was more prevalent among subjects with AOMS, whereas PPMS was more common among those with LOMS (Table 1).

DMT use was generally uncommon in both groups, as shown in Table 2. Among subjects with a history of RRMS (32 subjects with LOMS and 178 with AOMS) who had received DMT, those with LOMS discontinued treatment at an older mean age (64.0 years, SD 5.1) compared to subjects with AOMS (58.8 years [SD 6.7]; *p* = 0.04). The most frequently used DMTs were interferons and glatiramer acetate, and discontinuations were most often related to issues regarding disease course or adverse events. Notably, none of the subjects with LOMS had participated in a clinical drug trial, opposed to six subjects with AOMS who had.

**Fig. 2:**
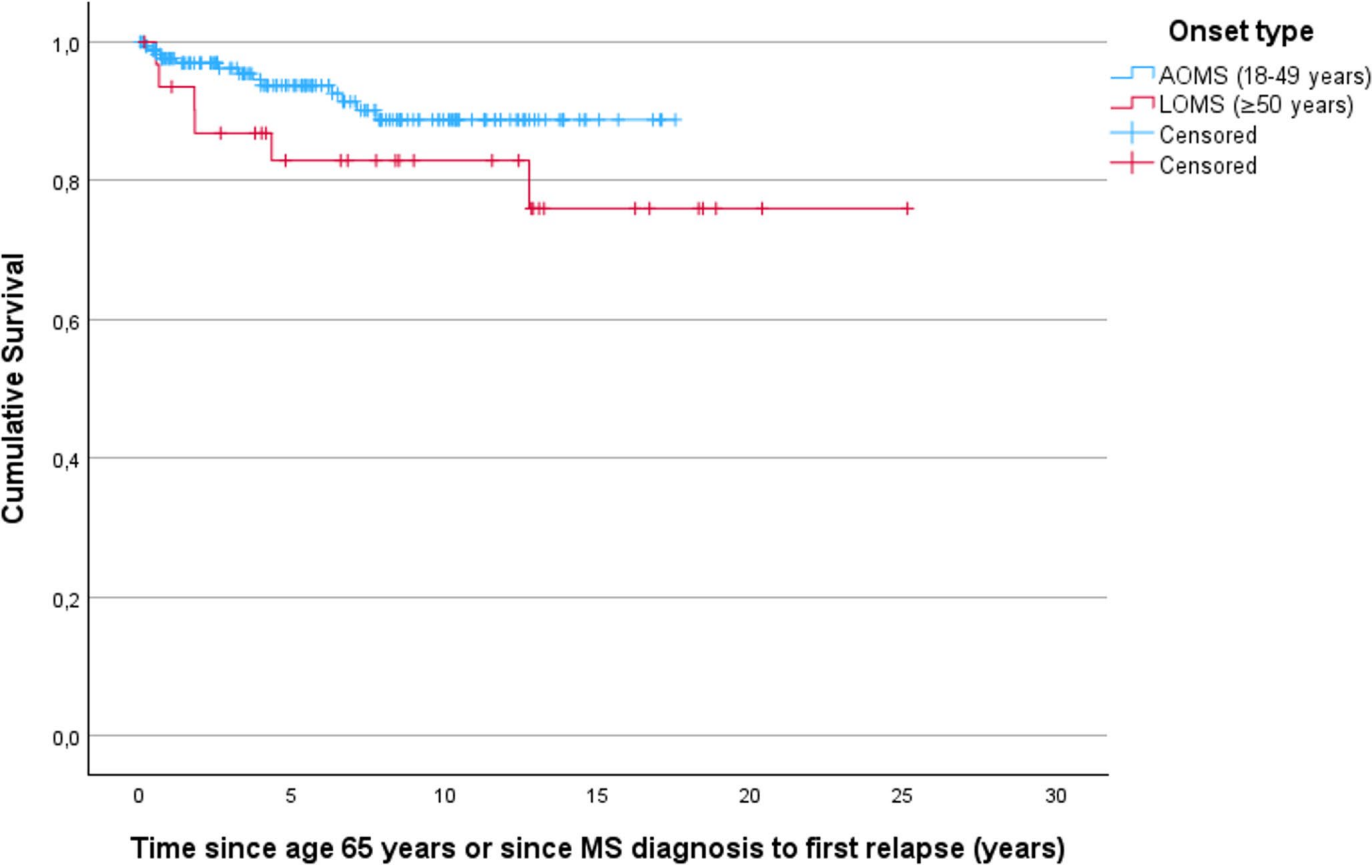
Survival curve displaying time in years from age 65 or MS diagnosis – whichever occurred last – until the first subsequent relapse. Censoring indicates reaching data cutoff date. A trend towards a shorter time to first relapse was seen in subjects with late-onset multiple sclerosis (LOMS) when compared to those with adult-onset multiple sclerosis (AOMS), but this was not statistically significant

Among subjects with a history of RRMS, relapses after age 65 occurred in 13/178 subjects with AOMS (7.3%) and 6/32 subjects with LOMS (18.8%, *p* = 0.05). After age 65 (or MS diagnosis, if last) ARR was low in both groups due to event sparsity (AOMS: mean 0.01, SD 0.03; LOMS: mean 0.02; SD 0.09, *p* = 0.05). Time to first relapse after age 65 (or MS diagnosis, if last) did not differ significantly between groups, although a trend towards a slightly shorter time to first relapse was observed among subjects with LOMS (HR 2.1, 95% CI 0.8–5.5, *p = 0.15;* Fig. 2). Out of 19 subjects who experienced a relapse after age 65, two subjects with AOMS and one subject with LOMS had experienced more than one post-65 relapse, while the remainder had only one.

After age 65, EDSS scores were available for 88/207 subjects with AOMS (42.5%) and 21/41 subjects with LOMS (51.2%). Although the last available EDSS score after age 65 was slightly higher among subjects with LOMS (median 6.5, interquartile range 5.5-7.0, *n* = 29) when compared to those with AOMS (median 6.3, interquartile range 5.0–7.0, *n* = 114, *p* = 0.046), median EDSS scores at specific age intervals (65–70, 70–75 and 75–80 years) did not differ significantly between groups.

**Table 1:** Demographic details of the study subjects

			AOMS		LOMS		Data available	*p*-value
							AOMS; LOMS	
			*n*	%	*n*	%		
Total number of subjects in the cohort			207	100	41	100		
Age, years			mean	SD	mean	SD		
		At disease onset	36.5	8.4	55.2	5.4	207; 41	***< 0.001***
		At diagnosis	43.7	9.9	56.8	7.0	207; 41	***< 0.001***
		At data acquisition	72.4	5.0	76.3	7.0	207; 41	***0.001***
Disease duration from onset, years			35.9	9.7	21.1	8.0	207; 41	***< 0.001***
Follow-up after age 65, years			7.4	5.0	11.3	7.0	207; 41	***0.001***
			n	%	n	%		
Sex								*0.33*
		Female	147	71.0	26	63.4		
		Male	60	29.0	15	36.6		
Smoking							155; 28	*0.67*
		No	91	58.7	19	67.9		*0.36*
		Yes	27	17.4	4	14.3		*0.69*
		Quit	37	23.9	5	17.9		*0.49*
CSF-specific oligoclonal bands			119	93.0	31	100.0	128; 31	*0.21*
Type of MS								
	Initial						207; 41	***< 0.001***
		RRMS	141	68.1	20	48.8		***0.02***
		SPMS	25	12.1	2	4.9		*0.18*
		PPMS	23	11.1	15	36.6		***< 0.001***
		Unspecified	18	8.7	4	9.8		*0.82*
	At data acquisition						207; 41	***< 0.001***
		RRMS	58	28.0	8	19.5		*0.26*
		SPMS	105	50.7	12	29.3		***0.01***
		PPMS	26	12.6	17	41.5		***< 0.001***
		Unspecified	18	8.7	4	9.8		*0.82*

AOMS, adult-onset multiple sclerosis; LOMS, late-onset multiple sclerosis; SD, standard deviation; CSF, cerebrospinal fluid; RRMS, relapsing-remitting multiple sclerosis; SPMS, secondary progressive multiple sclerosis; PPMS, primary progressive multiple sclerosis

**Table 2:** The use of disease-modifying therapies among subjects with a current or previous diagnosis of relapsing-remitting multiple sclerosis

		AOMS		LOMS	
		*n*	%	*n*	%
Subjects with a current or previous diagnosis of RRMS		178	100	32	100
Any history of DMT use		49	27.5	8	25.0
Current DMT use		1	0.6	0	
All DMTs used					
	Interferon	45	25.3	6	18.8
	Glatiramer acetate	16	9.0	2	6.3
	Fingolimod	2	1.1	1	3.1
	Dimethyl fumarate	1	0.6	1	3.1
	Teriflunomide	0		2	6.3
	Natalizumab	1	0.6	0	
Reasons for discontinuing last DMT^a^					
	A change in disease course	24	49.0	5	62.5
	Adverse events	8	16.3	2	25.0
	Stable disease course	8	16.3	1	12.5
	Inefficacy	2	4.1	0	
	Patient’s request	1	2.0	0	
	Unknown	6	12.2	0	

AOMS, adult-onset multiple sclerosis; LOMS, late-onset multiple sclerosis; RRMS, relapsing-remitting multiple sclerosis, DMT = disease modifying therapy; SD, standard deviation, ^a^Out of subjects who had any history of DMT use

## Discussion

In this real-world study, we analysed retrospective data from 207 older adults (aged ≥ 65 years) with AOMS (disease onset at age 18–49) and 41 with LOMS (disease onset at age ≥ 50) treated at TUH in Finland. Although the absolute event numbers were small, relapses after age 65 were numerically more common among individuals with LOMS when compared to those with AOMS. Additionally, we observed a trend towards a shorter interval from age 65 to first subsequent relapse in the LOMS group, although this finding did not reach statistical significance. Notably, multiple relapses after the age of 65 were rare in our cohort.

These findings suggest that although a greater proportion of individuals with relapsing LOMS may experience relapses at an advanced age, the risk of subsequent relapses after an initially relapsing disease onset seems to be low. Our observations align with those of Tremlett et al., who reported a more rapid decline in relapse rates among patients with older age at disease onset [9]. Currently, there is limited evidence regarding relapse risk specifically in older adults, as most previous LOMS studies have focused more on disability outcomes.

Some studies have already aimed at addressing this gap. Zinganell et al. found that although the overall ARR was lower among individuals with LOMS or VLOMS when compared to those with early- or adult-onset MS, aged individuals at diagnosis experienced more relapses than those with a longer disease duration [5]. In contrast, Mirmosayyeb et al. reported that individuals with LOMS more frequently experienced no relapses within the first two years post-diagnosis compared to those with earlier onset; however, their analysis did not stratify patients by disease course [25]. More recently, it has been emphasised that the most pronounced differences between AOMS and LOMS are observed in individuals with RRMS, highlighting the need to evaluate subgroups based on disease course in future LOMS studies [6].

The question of how actively individuals with LOMS should be treated remains a topic of considerable interest, particularly given the age-related decline in drug efficacy and the increased risks of infections and malignancies [14, 26]. In our cohort, DMTs use was infrequent compared to earlier reports [5, 6, 27]. A recent cohort study from Türkiye reported a shift toward second-line therapies, especially among individuals with LOMS [8]. We did not observe this trend in our cohort, where only four individuals had ever received high-efficacy therapies. In contrast, a large-scale Swedish registry study found no evidence that DMTs improve long-term disability outcomes in this population [6]. Supporting this, a meta-analysis concluded that none of the currently available DMTs demonstrated efficacy beyond a mean patient age of 53 years [14]. However, a recent report by Al-Araji et al. demonstrated that individuals with relapsing LOMS had similar treatment outcomes when compared to those with AOMS, challenging the assumption that older age should preclude aggressive treatment [15].

Considering patient age, the timing of DMT discontinuation needs to be brought into discussion earlier in the treatment course for individuals who initiate therapy for LOMS. In Finland, the national Current Care Guidelines, updated in 2024, suggest considering DMT discontinuation in individuals with stable RRMS after the ages of 55 and 60 [28]. However, the guidelines do not specify the duration of stability required before considering treatment de-escalation. In the DISCOMS trial, where the authors concluded that DMT discontinuation may be appropriate for patients over 55 years with stable MS, eligibility criteria included no relapses within the past five years or no new MRI lesions within the past three years [29]. Applying these criteria to older individuals with relapsing LOMS would result in many patients continuing DMT beyond the age of 60. In our cohort, patients with relapsing LOMS discontinued DMT at a significantly older mean age (64.0 years) compared to those with AOMS (58.8 years). Given the rising peak age of MS, the optimal timing for therapy in OAwMS remains to be determined.

Finnish neurologists generally adhere closely to the national Current Care Guidelines, ensuring uniform MS treatment throughout the country [28]. However, prior to the 2024 update, these guidelines did not include specific recommendations for managing older patients with MS. In the absence of age-specific guidance in the early 2000s, DMTs were rarely offered to patients beyond the age ranges represented in clinical trials. As MRI use was not routinely used as it is today, undertreatment may have occurred due to undetected subclinical disease activity. Furthermore, when the first high-efficacy DMTs became available, most individuals in our cohort were already older than 55 years. These factors likely contributed to the low overall use of DMTs, particularly high-efficacy therapies, in this population.

Future therapies may change the landscape of MS treatment. The Bruton’s tyrosine kinase inhibitor tolebrutinib was recently shown to reduce the risk of disability accrual compared to placebo in individuals with nonrelapsing SPMS aged 18 to 60 [30]. Still, additional analyses and trials are needed to further elucidate these findings. Therapeutic strategies targeting the neurodegenerative processes underlying MS progression would be extremely valuable, as disability accrual in pwMS seems to be disconnected from focal inflammatory activity [31].

Several studies have emphasised the progressive nature of LOMS [3, 5, 6, 27, 32–35]. In our cohort, the use of walking aids was common in both LOMS and AOMS groups after the age of 65. Median EDSS scores did not differ significantly between the two groups across the age ranges of 65–70, 70–75 and 75–80 years, but due to small sample size, the analysis was unlikely to detect small to moderate differences. Although our ability to assess disability progression was limited by infrequent follow-up in older age groups, our findings support previous observations that disability accumulation in pwMS is more closely associated with age than with disease duration [36], and that people with LOMS may reach certain disability milestones more rapidly than those with AOMS [6, 27].

The proportion of patients with LOMS in our cohort (16.5%) was consistent with previous literature, although the mean age of individuals with LOMS (76.3 years) was higher than that reported in earlier cohorts [3, 5, 8, 32, 34]. In line with prior findings, we observed that individuals with LOMS were more frequently diagnosed with PPMS, with a prevalence of 41.5% when compared to 12.6% among those with AOMS. Previous studies have reported the prevalence of PPMS among individuals with LOMS to range from 16% to 64% [6, 8, 27, 33, 34]. The high prevalence of PPMS in our LOMS subgroup may be attributable to their advanced age, as the likelihood of developing progressive MS appears to increase with age [3, 37].

The proportion of males in our study was slightly higher among individuals with LOMS (36.6%) compared to those with AOMS (29.0%), although this difference was not statistically significant. A similar trend of increasing male representation has been frequently reported in aging MS populations and LOMS cohorts [3, 5, 6, 27, 34, 35, 38]. In contrast, a Danish study offered an intriguing perspective, reporting that over a 60-year follow-up period, the incidence of MS – particularly LOMS – had doubled among women, while the increase among men was only moderate [17]. This shift was attributed to lifestyle changes among women, including reduced childbirth rates, increased prevalence of obesity and higher rates of cigarette smoking.

This study has its strengths and limitations. A key strength is the use of the Finnish MS registry to identify OAwMS. The registry is integrated into the patient information system and serves as a primary tool for neurologists during consultations with individuals with MS [39, 40]. It provides comprehensive data on all pwMS treated within specialised healthcare settings. However, it does not capture data from primary healthcare, limiting our ability to monitor disability progression after DMT discontinuation. In Finland, OAwMS who are no longer on DMT are typically managed by general practitioners.

Additional limitations include the small sample size, missing data from voluntary entries and limited MRI availability. MRI is often omitted in OAwMS due to the low likelihood of detecting active inflammation. EDSS scores at diagnosis were not included in our dataset, which restricted our ability to evaluate early disability trajectories. All individuals with a history of RRMS were included in our relapse analyses even if they had already transitioned to SPMS, which could introduce classification bias. Our findings regarding relapses, ARR and time to first relapse should be interpreted with caution due to event sparsity. The wide CIs for HRs for time to first relapse indicate that these findings should be considered exploratory. Since the robustness of our analyses are limited by the small sample size, replication in larger, more diverse cohorts is necessary to support broader conclusions.

In summary, our findings suggest that even though PPMS was particularly common among individuals with LOMS, those with relapsing LOMS may experience relapses even at an advanced age. Recent evidence considered, an individualised approach is recommended when caring for older adults with active MS. Since relapse activity declines with age, treatment discontinuation from currently available DMTs could be considered earlier in older individuals. Our findings underscore the importance of age-specific and holistic considerations in MS management, but due to the limited sample size, larger cohorts are needed to confirm these results. In addition to broadening the age range of clinical trials, we suggest that future studies should include subgroup analyses based on onset age and comorbidity, and incorporate a more comprehensive set of functional and cognitive measures in addition to MS-specific outcomes.

## Data Availability

Raw data cannot be shared openly due to data protection regulations.
